# Compression Behavior of Hybrid Tubes for Lightweight Steel Structures

**DOI:** 10.3390/ma14226797

**Published:** 2021-11-11

**Authors:** Gregorio Torres-Escobar, Juan Carlos Suárez-Bermejo, José Manuel Arenas

**Affiliations:** 1 Departamento Ciencia de Materiales, E.T.S.I. Navales, Univesidad Politécnica de Madrid, Av. de la Memoria n° 4, 28040 Madrid, Spain; g.torrese@alumnos.upm.es; 2 Centro de Investigación en Materiales Estructurales, Universidad Politécnica de Madrid, Calle del Prof. Aranguren n° 3, 28040 Madrid, Spain; juancarlos.suarez@upm.es; 3 E.T.S. Ingeniería y Diseño Industrial, Universidad Politécnica de Madrid, Ronda de Valencia n° 3, 28012 Madrid, Spain

**Keywords:** double steel tubes, non-metallic cores, design and manufacturing, compression test, strain energy

## Abstract

This research shows the results of an experimental investigation carried out on the compression behavior of hybrid steel tubes formed by two concentric steel tubes and four different fillers of non-metallic material interposed between both tubes: polyurethane foam, polyurethane, epoxy and a cement-based mortar. The tests show that the incorporation of a resistant filler in the double tube allows it to improve its mechanical behavior by allowing a second load cycle. Furthermore, the strain energy absorbed during the two cycles led to the conclusion that the epoxy-filled tube absorbed more energy per unit of weight than the other resistant fillers.

## 1. Introduction

The study of new hybrid materials is becoming increasingly more important in view of the need to solve problems in engineering applications where conventional materials do not offer adequate performance, that is, sufficient levels of strength and stiffness, ductility, reduced weight, etc. In some transport sectors, such as the maritime, railway, aeronautical, automotive and, in general, the civil and industrial world, weight optimization is a key factor.

Conventional materials have a number of limitations, such as having their strength and stiffness linked to their density and, consequently, to their weight. Hybrid materials, on the other hand, allow for the design of different elements through the combination of materials with different characteristics. At the same time, they allow for the introduction of manufacturing techniques in their design that maximize the capacity of each material in the most efficient way, such as the orientation of fibers in the direction of the principal stresses, the use of materials at different temperatures or in different states during manufacturing, the arrangement of the materials for better durability, efficiency, etc. 

These materials also provide other characteristics in addition to the strength–weight ratio [1], such as better performance against nonlinear phenomena such as buckling and impact [2], with better ductile behavior [3,4,5,6,7,8], blast loading [9], fire resistance [10,11,12,13]. 

Hybrid materials have the added difficulty of joining different elements [14] and the interface between the materials that make up the hybrid element itself, which must be studied in depth to ensure that the design and materials work together throughout their use.

In addition, the analytical determination of the behavior of these types of materials is more complex, as it is necessary to combine experimental studies with numerical analysis.

The study of compressed elements is also complex due to the instabilities that occur above certain load levels. These instabilities are known as buckling and can occur globally, locally, or as a combination of both [15]. Buckling depends on the relationships between certain parameters, such as thickness, width or diameter, and length of the elements, as well as on the type of section, if the section is open or closed, its imperfections, and the type of material used. 

This research studies a series of hybrid elements subjected to compression in order to determine their ultimate strength and identify possible failure modes. To this end, elements consisting of two concentric steel tubes and a circular cross section CHS (circular hollow section) were used, between which a resistant core of non-metallic material was incorporated. Tubular sections are known to have particularly good buckling behavior [1,15,16] and circular tubular sections are the most efficient against the effects produced by the pressure of confined materials [6,17].

There are other works in this field that introduce some variant in the design of hybrid elements, such as an outer and inner tube made of aluminum [18] or stainless steel [19], different cross sections (CHS—RHS) in the two metallic tubes that make up the same hybrid element [20], and elements designed with a slight taper [21] that allow conclusions about the effect of the inclination angle; others focus on the slenderness of the elements [22,23]. Likewise, other works [24,25] have studied the effect of the core on different grades of concrete strength. 

The present research aims to broaden the range of experiences necessary to obtain a more reliable knowledge of these types of elements. Some particular variables were considered when designing the configuration of the tested tubes, such as the type of steel for the CHS tubes, the ratio between the diameter and the thickness of their walls, the core material, and its thickness (which depends on the difference in diameter of the skins). Specifically, this study is focused on testing tubular elements with medium slenderness and with a Class 1 section classification according to Eurocode 3, EN 1993-1-1 [26].

Thus, the purpose of this research is to show the experimental results obtained in the compression tests of four hybrid tubes with different non-metallic cores. The conclusions obtained will improve the knowledge of these types of hybrid tubular structures and thus facilitate their better and wider use in the construction of lightweight and resistant structures subjected to compression.

## 2. The Design of Hybrid Tubular Elements

In this work, a method for the analysis of the CHS hybrid tubes, consisting of two concentric tubes of circular cross section and a core of non-metallic material that filled the space between the two tubes, was developed. This methodology included the analysis and comparison of the results obtained in the different proposed configurations. The aim was to check the failure mode of the tubes and to study their behavior during the test by analyzing different parameters.

To understand the behavior of the steel tubes and to test each of the configurations used, the regulations regarding the design of metallic structures were followed and analyzed, using mainly Eurocode 3 in its different parts, EN 1993-1-1 [26] and EN 1993-1-6 [27]. 

EN 1993-1-1 [26] establishes four types of cross sections [28,29,30,31], with Class 1 being the most robust or compact and Class 4 being thin-walled, that is, having a higher diameter–wall thickness ratio. 

Class 1 sections have sufficient rotational capacity to allow for plastic global analysis. Class 2 sections have a lower rotation capacity, only partially allowing the plastic moment resistance of the section to be mobilized. Class 3 sections are limited in their resistance capacity to the elastic moment of the section; in these sections, the ultimate moment of resistance is reached when one of the fibers of the section reaches the elastic limit of the material. Finally, class 4 sections can only partially mobilize the elastic moment when the plates forming the section have previously buckled. As a consequence of the instability phenomenon in Class 4 sections, the design codes [16] require the use of a reduced effective section to obtain the maximum moment. 

In the particular case of CHS and Class 4 sections, Rotter [29] and Marco [32] propose applying EN 1993-1-6 [27]. Alternatively, different researchers [1] have used the German standard DIN 18800-4 [33] for the same purpose. 

The ultimate theoretical load of the hybrid tube can be determined using a procedure similar to that described in Eurocode 4 [34], considering the inner tube as part of the reinforcement [24]. The standard BS5400 [35] also proposes a similar procedure to obtain the maximum load [11,23]. Similarly, the energy absorbed during the compression test can be obtained according to the method described by Chung et al. [2].

The choice of a particular cross section type is a design constraint that has implications on structural behavior. The minimum wall thickness depends not only on the allowable stresses in the walls, but must also guarantee the buckling resistance of the plates. Occasionally, thin-walled sections cannot be avoided because some industrial applications require large diameters compared to those of the cross section. For example, Schaumann et al. [1] used these hybrid elements in wind turbines with large diameters and steel thickness that is within the nominal manufacturing values. 

**Figure 1:**
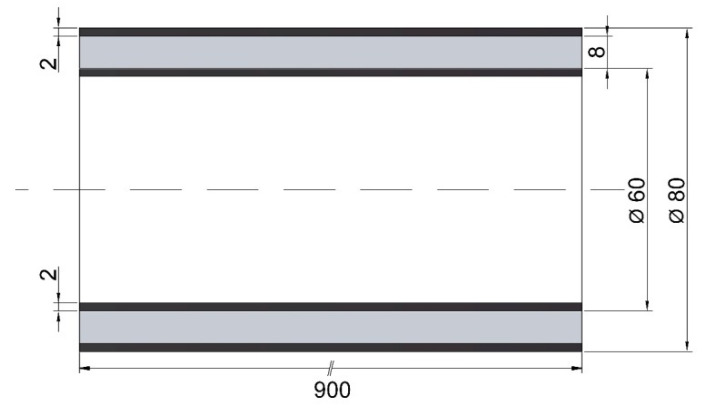
Hybrid tubular element: outer tube, inner tube, and core filled with different nonmetallic materials.

In this research, hybrid CHS tubes of the same S275JR steel with a length (L) of 900 mm have been considered. The outer diameters of the selected steel tubes are 80 and 60 mm and they have a skin thickness of 2 mm. (Figure 1). With these characteristics, the tubes are considered in the medium-slender range (λ) and with a Class 1 cross section according to Eurocode 3 [26]. With this configuration, the thickness of the non-metallic core of the hybrid elements is 8 mm.

## 3. Experimental Methodology

### 3.1. Selected Materials

Four materials have been used to fill the core of the hybrid elements:

1. Low-density polyurethane foam with 15 kg/m^3^.
2. Two-component hydrophobic polyurethane grout (Elastopack^®^ 201, De Neef Technologies (Barcelona, Spain)).
3. Two-component thixotropic epoxy (Multitek^®^, De Neef Technologies).
4. A single component cement-based mortar with very high mechanical strength (SikaGrout^®^-295 by Sika (Baar, Switzerland)).

Table 1 shows the characteristics of the four hybrid elements tested. 

### 3.2. Manufacturing of Tubular Hybrid Elements

The manufacturing of the hybrid tubes consisted of cutting, assembling, and placing the core material. In the case of polyurethane foam, it was injected at low pressure with a cannula. Epoxy (Elastopack-201) and polyurethane elastomer (Multitek) based composites were filled by pouring because the mixture had good fluidity. Finally, the mortar (SikaGrout-295) had to be kneaded with the appropriate dosage and the filling process was carried out by vibrating the system to ensure good compaction of the mixture. The tests were carried out 30 days after manufacturing to ensure that the cores were hardened.

**Figure 2:**
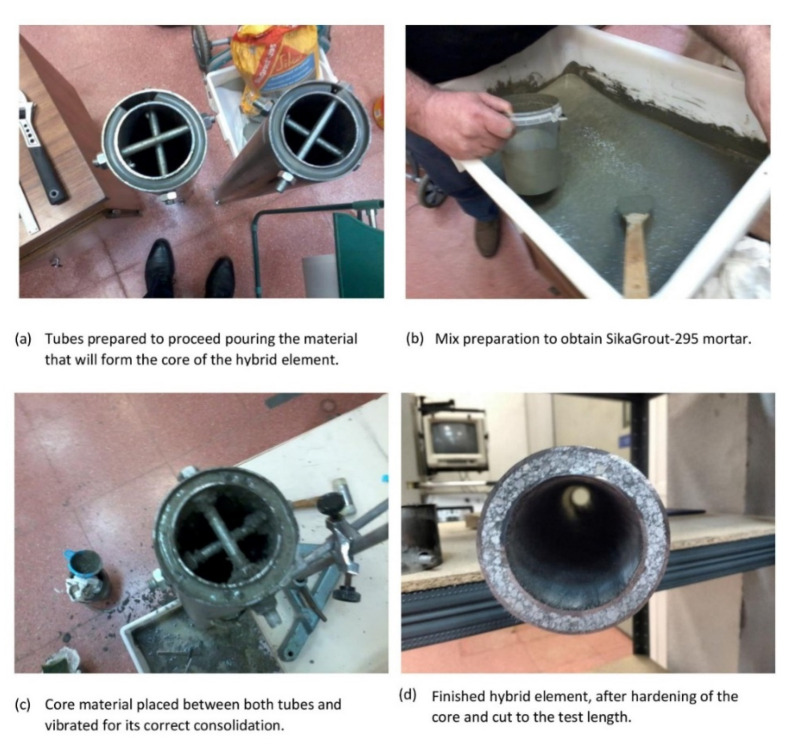
Manufacturing of a hybrid element with mortar core: (**a**) Preparation of steel tubes, (**b**) Kneading of SikaGrout-295 filler material, (**c**) Filling of filler material, (**d**) Finished hybrid element.

After the core material hardened, two plates were placed on both ends of the tubes. These plates were fixed at the cross-welding points to guarantee perpendicularity with respect to the directrix of the tubes and thus ensure correct load transmission. Figure 2 shows the manufacturing process of the SikaGrout-295 cement-based mortar core element.

### 3.3. Instrumentation of the Test Elements

The tests were carried out at the Centro de Investigaciones de Materiales Estructurales (CIME). These tests consisted of the application of a quasistatic axial load using a custom-built Servois universal machine of 120 t capacity (Servois, Pinto, Spain), installed in 2014. Measurements of the applied load and displacement were taken in the direction of load transmission. Images of each of the tests were recorded with a 2009 Sony Handycam video camera (Sony, Tokyo, Japan).

The VIC 2D/3D digital image correlation program (Correlated Solutions inc. Irmo, SC, USA, v.2010) from Correlated Solutions was used for the measurement and analysis of deformations. Image capture for transmission to this program was performed with an AVT Dolphin F201B camera (Allied Vision Technologies GMBH, Stadtroda, Germany) with the sensor: Type 1/1.8 Progressive Scan b/w SONY IT CCD, 1628 (H) × 1236 (V) pixels and lens: Pentax, focal length 75 mm, maximum aperture f/2.8. This system was used to obtain strain maps (µm/m) in the central third of the element tested, for both longitudinal units (y) and transverse units (x). A map with the vectors indicating the deformation flow was also obtained.

**Figure 3:**
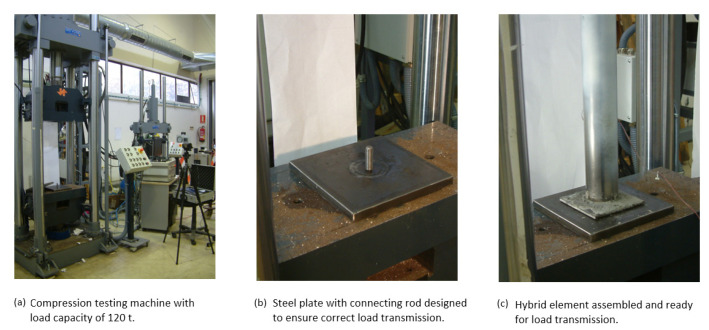
Preparation of the compression test of the hybrid elements: (**a**) Servosis 120 t universal testing machine, (**b**) load transmission plate with connecting rod, (**c**) assembled hybrid element assembled ready for load transmission.

**Figure 4:**
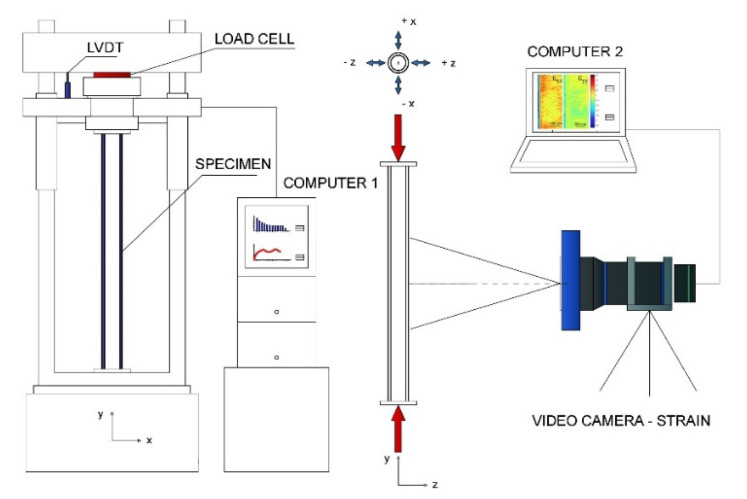
Diagram showing the general layout of the tests performed.

The load transmission end plates, which were joint at both ends of the tubular element, were designed with a hole that allowed passage for a rod that connected to the clamps of the test machine. This type of joint prevents movements in any direction perpendicular to the axis of the tube; however, at the same time, the width of the hole with respect to the rod is sufficient to guarantee rotation and does not cause embedment. Figure 3 shows the images of the fixture on the test machine. Figure 4 shows a diagram of the general layout of the tests.

### 3.4. Compression Tests of Hybrid Tubes 

Before the tests were performed, the verticality of the tubular element was verified and checks were made to ensure that all sensors were calibrated and synchronized correctly. The deformation camera was used to carry out preliminary tests with other tubes to verify that everything was working correctly and that the distance of the deformation camera was sufficient to obtain good quality values.

The applied load speed was 0.2 mm/s to avoid generating dynamic effects. As the load increased, the hybrid element mobilized its resistance according to its stiffness, reaching a maximum load that caused the tube to lose its stability, thus terminating the test or, in contrast, admitting another load cycle. If the tube did not lose stability after reaching the maximum load, this was because the tube had absorbed energy from the uniform crushing of the section without losing its verticality through global buckling. During this crushing, the load decreased as a result of the transformation into strain energy. Subsequently, the element regained stiffness and the load increased again to a new maximum load.

Once the tests were completed, all records were checked to ensure that they had been properly recorded and that there had been no loss of information.

## 4. Results

**Figure 5:**
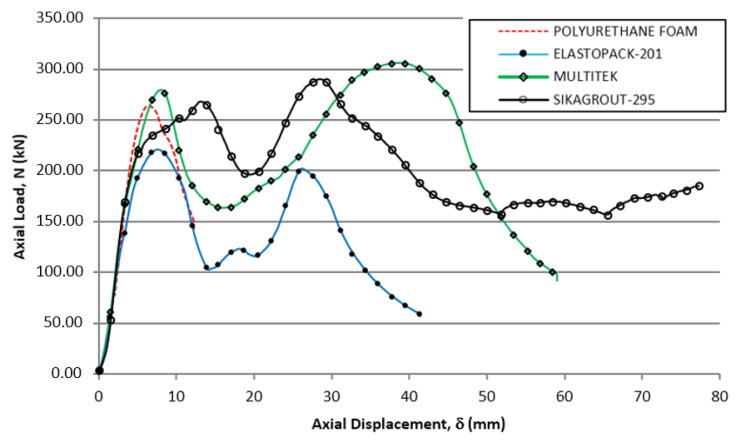
Hybrid tube load-displacement curves in compression tests and single steel tubes used as inner tubes in the hybrid element.

During the test, measurements of longitudinal ε_y_ (mm/m) and transverse, as well as displacements v_x_ (mm) and v_y_ (mm), were obtained with the deformation camera in the central zone of the element. The testing machine generated the applied load N (kN) according to the displacement δ (mm) in the longitudinal direction. In Figure 5 the load values obtained are plotted according to the total deformation of the element in the direction of its axial axis.

Displacement (δ) may have been due to either a purely elastic deformation of the element or a shortening due to a local deformation by plastic failure. Once the tube began to bend and, as it was bending, buckling failure occurred suddenly.

As can be seen in Figure 5, all tubular elements had had two maximum load cycles before failure, except the tube made with a polyurethane foam core, which reached its capacity limit by buckling in the first load cycle. Likewise, the element with the mortar core (SikaGrout-295) was the only one that did not collapse due to global buckling after the maximum load had been reached in the second cycle. In this case, although the tube still retained its load capacity with a displacement of 80 mm, the test was terminated because the crushing deformations at the top of the tube were considered significant. Elastopack-201 and Multitek manufactured elements underwent global buckling when they reached maximum load in the second load cycle.

Table 2 shows the results obtained during the tube compression tests. Thus, for each cycle and tube type, the axial load, displacement, and time are registered. 

**Figure 6:**
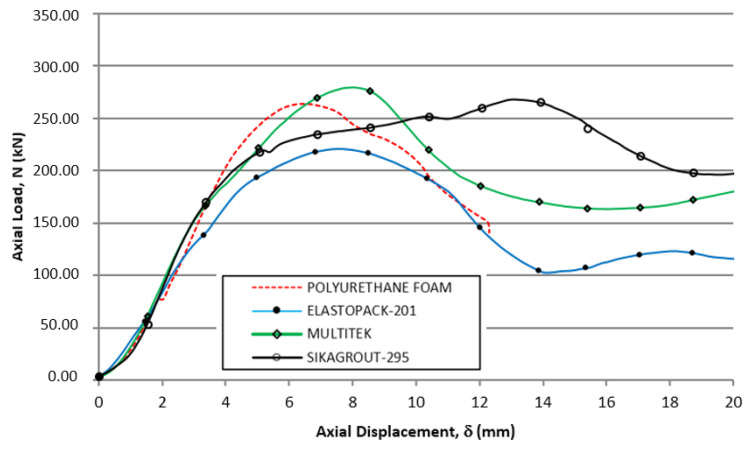
Load-displacement curves of the elements in the first load cycle (abscissa: origin—20 mm), from the beginning of the test until the elements are unloaded after the first maximum load.

In Figure 5, the first zone, the behavior of the four hybrid tubes was very similar between 0 and 15 mm of absolute displacement. First, there was a load increase with a certain almost identical slope (corresponding to the compression stiffness of the tubular element). After, a maximum load above 250 kN was reached (somewhat lower for the case of the Elastopack-201 filler). Finally, there was a sharp drop in the load to a lower level (which is different for each tube). This drop was caused by crushing the section of the tube that was closest to the load introduction plate, producing local plastic deformation in that area, while the rest of the tube remained intact. Figure 6 shows, in detail, a similar behavior for the four hybrid tubes during the first 20 mm of axial displacement.

In the tubes that did not buckle during this first cycle, the decrease in load was caused by crushing the area of the tube closest to the load introduction plate, producing uniform plastic deformation throughout the section in that area, while the rest of the tube remained intact.

The polyurethane foam-filled tube (the filler that did not provide any resistance characteristics) failed prematurely by buckling in the test, since its central section was not able to withstand the bending stress that occurred when the critical load was reached. In contrast, in the other three types of tubes, the filler provided enough stiffness in the central zone to increase the bending capacity and prevent the tubes from losing their stability due to buckling.

The three tubular elements that did not buckle after the maximum value of the first cycle underwent a second load cycle starting from the lowest point of each of the curves and reaching a new maximum load in the second cycle. At this point, the elements had yielded between 14 and 20 mm, which was enough for the steel tubes and fillers to become overloaded as a single joint element since the beginning of this second cycle.

Table 3 shows the maximum values for each of the cycles and the minimum values that occurred when the hybrid tube yielded. With these values, two efficiency coefficients were obtained which, through the ratio between the minimum and maximum values, show how much the load was reduced after reaching each of the maximum loads in cycle 1 (η11) and cycle 2 (η22). Furthermore, another coefficient, which shows the relationship between the maximum value of cycle 1 and the maximum value of cycle 2 (η12), was included.

It can be seen that in the case of cycle 1 (η11), in all the elements, the load yielded at values between 50 and 60% of the maximum load reached, and in the tube with the SikaGrout-295 filler, 73% of the load was maintained. In cycle 2 (η12), in the three elements, where no buckling occurred after reaching the first maximum load, the load yielded after a peak of between 20 and 30%, and the SikaGrout-295 elements maintained 54%.

The efficiency coefficient between the maximum loads of cycle 1 and cycle 2 (η12) shows that in the tube filled with Elastopack-201, the load increased by 9% in cycle 2. In the rest of the hybrid tubes, except for the polyurethane foam tube, which had only one cycle, the maximum values decreased by 10% in the second cycle.

**Figure 7:**
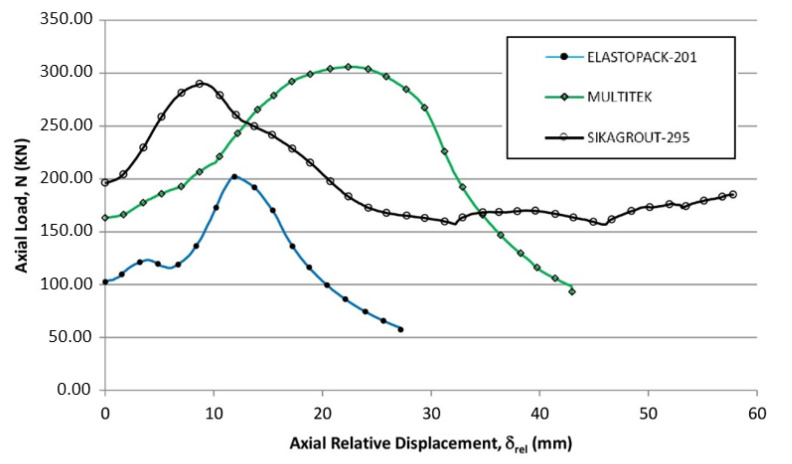
Load versus relative displacement curves of the tested elements from the second load cycle (abscissa: “N_1,min_” values of each of the tubes have been synchronized at the origin—δ_rel_). The unloading points are matched at the origin and the displacements are the increases over the departure time.

To better visualize the behavior of the three tubular elements in the second cycle (Elastopack-201, Multitek, and SikaGrout-295), a graphic representation was made (Figure 7) in which the three curves have been brought to a common abscissa origin and the increase in deformation in all tubular elements is indicated from this common origin.

As such, the zero abscissa value in Figure 7 corresponds to the value of 14.2 mm for tubular elements with Elastopack-201, 16.1 mm with Multitek, and 19.6 mm with SikaGrout-295, with respect to the origin of the test (Figure 5 and Figure 6). The tubular element with SikaGrout-295 sustained a load of 196.30 kN at the origin of the abscissa axis, 163.24 kN for the element with Multitek, and 102.61 kN for the element with Elastopack-201.

During the second load cycle, the elements filled with Multitek and SikaGrout-295 improved their resistance with respect to the previous cycle, with values of 305.97 and 298.18 kN, respectively. In the case of Elastopack-201, it reached a value of 202.18 kN, which is somewhat lower than the value reached during cycle 1. Except for the tube filled with SikaGrout-295, the rest of the elements ultimately lost stability due to buckling. The SikaGrout-295-filled tube, after reaching a maximum load in cycle 2, yielded without buckling to a new minimum value of 156.96 kN and began to accept more load until reaching a value of 185.11 kN. At this point, an absolute displacement of 77.35 mm had occurred in a non-uniform manner, producing a shift towards one of the sides; for this reason, the test was terminated.

**Figure 8:**
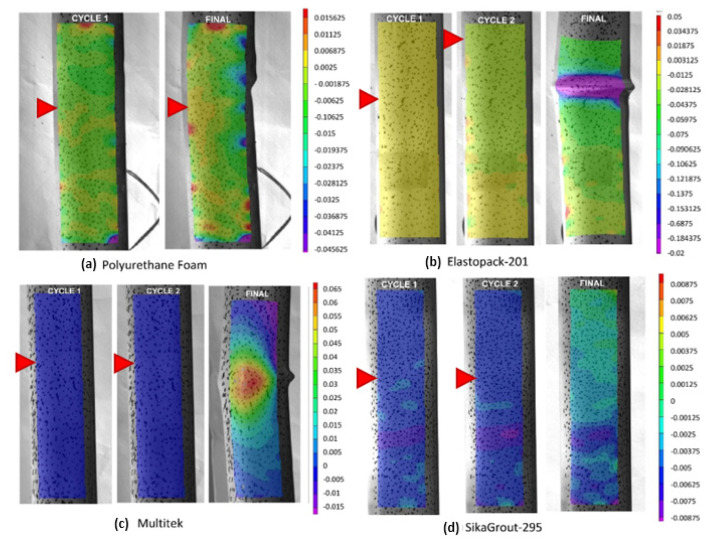
Deformations ε_y_ (μm/m) at the maximum load of cycle 1 and cycle 2 and at the end of the test in each of the hybrid elements tested; core filler materials: (**a**) Polyurethane foam, (**b**) Elastopack-201, (**c**) Multitek and (**d**) Mortar (SikaGrout-295). Red arrows indicate loci of maximum strain at peak loads.

Figure 8 shows the deformation maps ε_y_ (μm/m) obtained by the digital image correlation system at the maximum load of cycles 1 and 2 and at the end of the test. In general, at the beginning of the test, the observed strain distribution was uniform in all elements. From a certain load level, near the maximum loads, a certain loss of uniformity in the strain distribution was detected, but the values maintained the same sign when following the compressed sections. Once the extreme fibers of the same section yielded values of the opposite sign, the element began to bend; subsequently, buckling occurred.

In the polyurethane core element, longitudinal deformations maintained a certain uniformity up to the maximum load obtained during cycle 1. Once this value was reached, the strain distribution showed opposite sign values as a consequence of the bending and global buckling that occurred. Similarly, this happened in the Elastopack-201 and Multitek core elements, the difference being that the buckling occurred in cycle 2.

**Figure 9:**
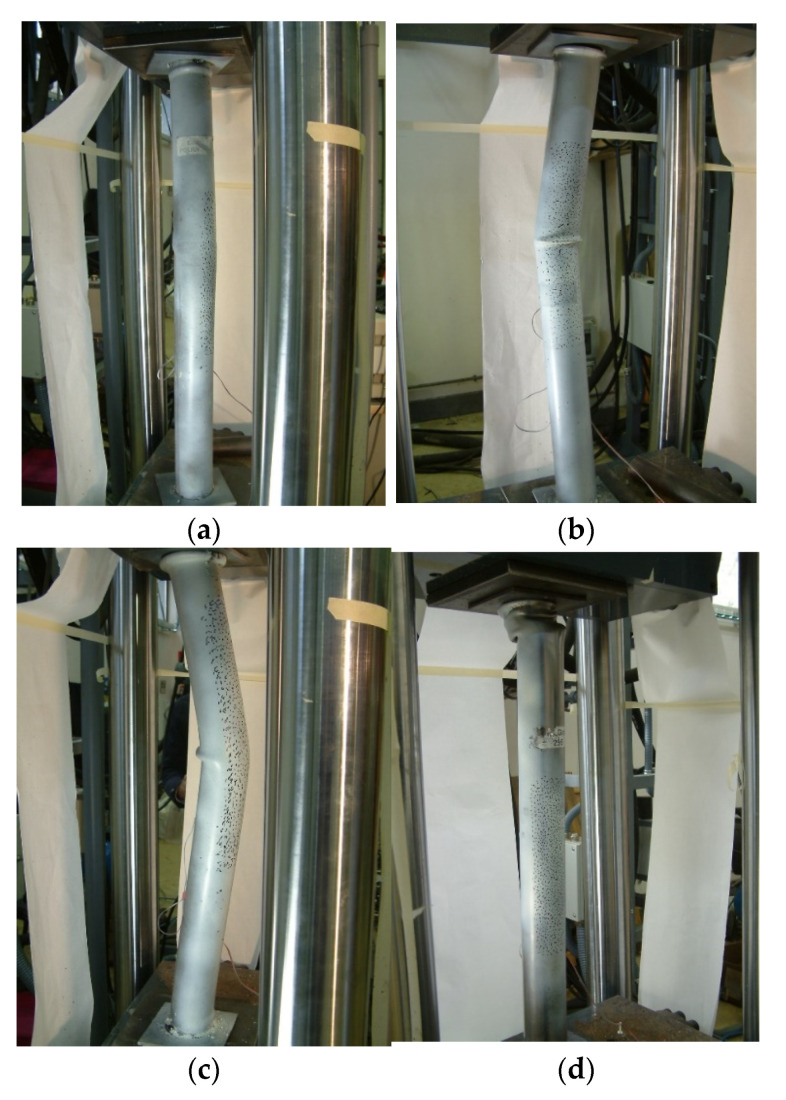
State if hybrid tubes at the end of the test and the cycle in which the state is produced. Core filler materials: (**a**) Polyurethane foam–element with a polyurethane foam core, buckling in cycle 1, (**b**) Elastopack-201–element with Elastopack-201 core, buckling in cycle 2, (**c**) Multitek–element with a Multitek core, buckling in cycle 2, and (**d**) Mortar (SikaGrout-295)–Element with SikaGrout-295 core, no loss of stability due to buckling.

Figure 9 shows the condition of the hybrid tubes at the end of the test. The mortar core element (SikaGrout-295) maintained a practically uniform deformation distribution at the end of the test, as there was no global buckling of the element. As can be seen in Figure 9d, all deformations were due to crushing of the upper part. Throughout the test, the mortar tube consistently maintained a load above 160 kN, with a tendency to increase toward a third cycle when the test was completed (with a load of 185 kN).

**Figure 10:**
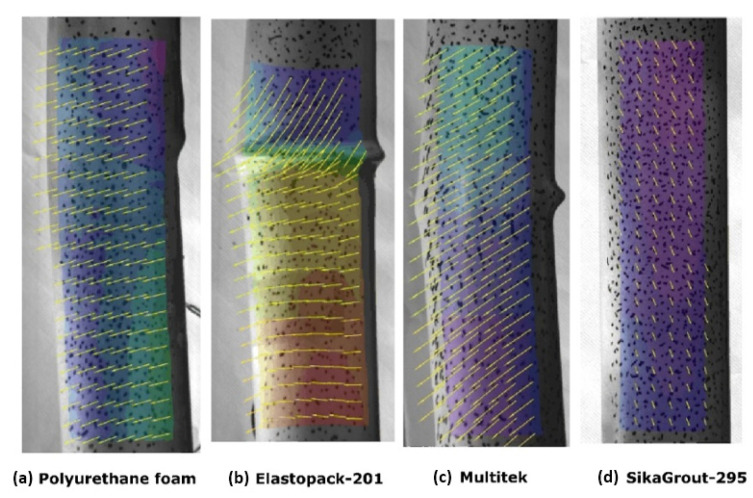
Deformation vector flow at the end of the test; Core filler materials: (**a**) Polyurethane, (**b**) Elastopack-201, (**c**) Multitek, and (**d**) Mortar (SikaGrout-295).

Using the correlation of transverse (ε_x_) and vertical (ε_y_) deformations, a particularly useful map was created to observe the direction of the resulting flow (Figure 10). As the element had equal inertia in all diameters, and since the camera was located orthogonally to the compression testing machine, once the element began to deform laterally by buckling, it was free to do so in any direction and was not guaranteed to coincide with the plane of the camera shot. For this reason, quantitative values of ε_x_ and ε_y_ cannot be considered, but can be used as a qualitative indicator of the direction of the deformation vector flow.

In Figure 10, it can be seen that at the end of the test, in the elements that buckled globally, the flow acquired a rather pronounced horizontal component. In the case of the mortar-made element (SikaGrout-295), since no buckling occurred, the flow maintained a greater vertical component.

## 5. Discussion

The hybrid tube filled with polyurethane foam, a filler material that does not provide compressive strength and is used as a simple filling for the initial placement of the tubes, achieved a high load level without experiencing separation of the tubes or any decoupling effect of the two tubes. Finally, once the joint behaved as a single element, it failed by buckling after reaching the maximum value in the first load cycle. The chosen tube configuration (thicknesses, diameters, and spacing between them) showed that with a non-resistant filler, there was no destabilization for early loads as a good load capacity was reached.

The Elastopack-201 filler is an elastomer that, due to its constitution, allows for significant deformations when a load is applied in a given direction. As a consequence of this, significant lateral deformation (coherent with the direction of the load application) is generated as the material has a significant Poisson ratio (quasi-incompressible). Additionally, this material has a low longitudinal deformation mode compared to other more compact materials, and this causes it to experience instantaneous deformation when it undergoes early loading. As the compressive load on the element increases, the core material is compressed, and the lateral reactions of the core against the tube walls are greater. The contribution to compression expected from the constrained elastomer between the two tubes is due to the fact that the lateral reactions it generates are impeded by the walls of the tubes; thus, the core material shows greater opposition to deformation in the direction of load application, increasing its resistance. As these reactions become greater (as the compressive load on the tube increases), there is a point at which the hybrid tube is destabilized by thrusts and loses its load capacity. Due to this phenomenon, the Elastopack-201 tube reached a load lower than that obtained with the polyurethane foam tube. After the first maximum load was reached, the tube yielded without buckling, with buckling failure occurring at the second maximum peak.

However, Multitek and SikaGrout-295 are materials with high elastic moduli (see Table 1) and are capable of absorbing large loads without experiencing large deformations in the early stages of loading. Consequently, these materials do not have appreciable deformations for low loads. These begin to occur when the loads are high. On the other hand, since these materials have a low Poisson ratio (around half that of Elastopack-201 and similar to that of steel), these lateral deformations produce smaller thrusts against the tube walls, which are significant for high load levels. Unlike Elastopack-201, these fillers have contributed to increasing the compressive load capacity of the hybrid element, preventing the element from destabilizing under early loads.

Finally, after the maximum load was reached in the second cycle, the Multitek-filled element failed by buckling, with a value greater than the maximum load reached in the first maximum peak. The element filled with SikaGrout-295, even with significant deformations after reaching the maximum load of cycle 2, maintained stability until the end of the test. As can be seen in Figure 9d, the deformations in the upper part of the element with SikaGrout-295 were significant when the test was completed.

Table 4 shows the values for the weight of the elements, the cost of the tubes taking into account the fillers used, and, based on this, the relationships between the maximum loads for each of the cycles and these values. This produces load efficiency values in relation to weight and cost.

The element that withstands the highest ultimate load among the tubes with two load cycles was the Multitek filled tube (N_2,max_ = 305.97 kN). Compared to the tube filled with polyurethane foam, a filling that does not provide resistance, this value was 16% higher.

The tube with the highest final load–weight ratio obtained was the tube filled with polyurethane foam (N_1,max_/weight = 4.45 kN/N) and its ratio was 56% higher than the tube with the lowest ratio (SikaGrout-295) in the first cycle (2.86 kN/N), which obtained a value very similar to Elastopack-201 (2.94 kN/N). During the second cycle, the tube with the highest maximum load–weight ratio obtained was the tube filled with Multitek (N_2,max_/weight = 3.54 kN/N). This was a difference of 32% with respect to the Elastopack-201-filled tube, which had the worst ratio with 2.69 kN/N.

The element with the best ultimate load–cost ratio was the tube filled with polyurethane foam (N1, max/cost = 14.55 kN/Eur), which saw a difference of 635% with respect to the lowest ratio, the tube filled with Multitek. During the second cycle, the tube with the best ultimate load–cost ratio was the SikaGrout-295 tube (13.21 kN/Eur), which was 508% higher than the Multitek filled tube (2.17 kN/Eur), which had the lowest load–cost ratio in the second cycle.

Among the tubes that withstood two load cycles, the element with the SikaGrout-295 core had the best combination of maximum strength and maximum load–weight and maximum load–cost ratios. In terms of maximum strength and load–weight ratio, the Multitek-filled tube showed better results, but did not show any significant differences with respect to the Sikagrout-295 tube. On the contrary, the Multitek tube had a very poor cost–efficiency ratio.

The strain energy, a consequence of the internal energy consumed during the test, was also an indicator that allowed a comparison of the elements tested and an understanding of their degree of ductility. In terms of energy, the stable or unstable equilibrium of a system can be defined through the potential energy of the system. The existence of a minimum value of energy guarantees stability up to a certain limit at which collapsing occurs. In hybrid elements, it is complex to theoretically obtain this strain energy. However, in this study, it was possible to determine the strain energy of the hybrid tubes from the experimental results obtained in the tests that had been carried out.

**Figure 11:**
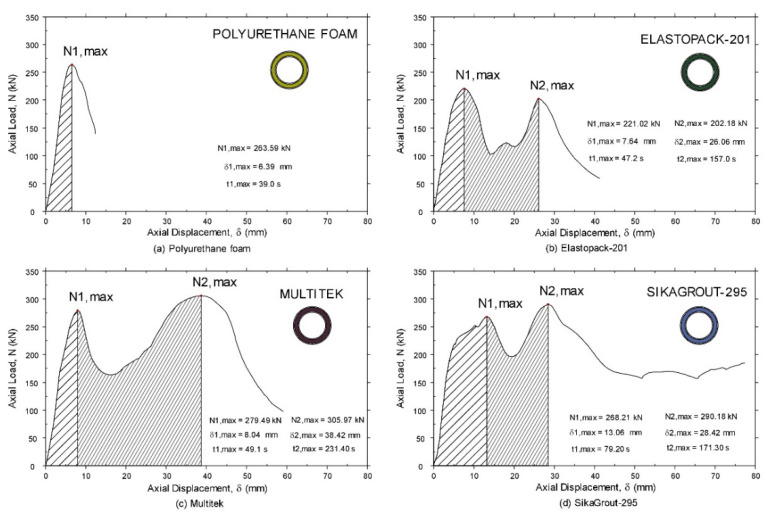
The strain energies of the elements represented by the area enclosed under the load-displacement curve: (**a**) Polyurethane foam, (**b**) Elastopack-201, (**c**) Multitek and (**d**) Mortar (SikaGrout-295).

Figure 11 shows the load-displacement graph, with two distinct shaded areas. The strain energy reached at the maximum load of cycle 1 (N_1,max_) was obtained from the area enclosed under the curve once the value was reached. Similarly, the strain energy reached at the maximum load of cycle 2 (N_2,max_) was obtained in the same way, in the latter case being the sum of the two areas differentiated in the images.

Table 5 shows the strain energy values obtained in each of the cycles (E_a1_ and E_a2_) and the ratios of these values (E_ar1_ and E_ar2_) to the weight of each of the elements.

Elastic moduli of the core materials are much lower than the modulus of steel. Consequently, the overall stiffness of the hybrid tubes is not very dependent on the core-specific material (see Figure 5). However, elastic moduli play an important role when approaching the buckling load because the core acts as a supporting scaffold to prevent premature collapse of the steel walls. The post-buckling behavior is also very sensitive to the specific value of the core material stiffness and is responsible for extending the load history of the tubes, with a recovery of the load capacity, up to a point where a second peak is reached and final failure occurs.

During cycle 1, the element with the SikaGrout-295 core absorbed a higher amount of relative energy, with a value of 27.46 J/N. During cycle 2, it was the element made with a Multitek core that absorbed the highest relative energy, with a value of 96.10 J/N. Hybrid elements that absorbed the most strain energy, both in absolute terms and in relation to their weight, succeeded in delaying the failure of the element for the longest time (for the same rate of loading).

A more ductile element has a greater capacity to absorb deformations within the elastic–plastic range without losing its overall stability. The degree of ductility in compressed elements is related to the shape and dimensions of the cross section and the properties of the material used. In simple steel elements, high-strength steels allow for a higher load capacity, but the ductility is compromised.

It is important to note that there were differences in the load-displacement curves, and this was reflected in the energy absorbed by each of the elements. The initial slopes in the diagrams were very similar as a result of the fact that the steel tubes initially absorbed a greater amount of load. This happens in systems with several elements that structurally work together. The elements with more stiffness are those that react proportionally to the load, and when they begin to yield, even within the elastic range, the other parts with less stiffness begin to contribute. In the case of hybrid tubes, the contribution was different because the material is confined between the two steel tubes. As such, the polyurethane foam filler did not contribute structurally but did not damage the hybrid element, as it did not destabilize the structure with lateral thrusts. The viscoelastic material filler (Elastopack-201) generated instability by allowing large deformations (lateral thrusts) from the beginning and the last two of the materials considered (Multitek and SikaGrout-295) allowed for the load capacity without damaging the stability of the hybrid element, due to their greater stiffness and their type of internal structure.

The difference in behavior between SikaGrout-295 and Multitek fillers lies primarily in the ability of the former, which is a cement-based mortar (microconcrete), to allow gradual adjustment as microcracks occur. This allowed it to absorb more energy in the first load cycle by allowing a smoother slope (yielding more gradually) up to the maximum load of the first cycle. In the case of the Multitek tube, which has an epoxy resin base, the filler structure remained stiff (without gradual yielding) until a more sudden breakage of the material occurred. During the second Multitek cycle, the tube absorbed more energy than the SikaGrout-295 tube. However, it should be noted that this element started to increase the load for the second cycle from a lower load value than that of the SikaGrout-295 tube (163.24 kN vs. 196.3 kN).

Table 6 summarizes the results and discussion of the tests carried out, indicating the percentage difference in maximum load (for the first and second cycles), the load–weight ratio, and the deformation energy. In relation to the maximum load, the element filled with Multitek is the most resistant in the first cycle, with 26.5% more than Elastopack-201, while in the second cycle its resistance is 51.3% higher than this. Regarding the load–weight ratio, the polyurethane foam filled element is 55.6% better than the SikaGrout-295 filled element in the first cycle, while in the second cycle the Multitek filled element is the one with the best ratio, with a 31.6% higher load–weight ratio than Elastopack-201. Finally, the element filled with SikaGrout-295 is the one that absorbs more energy in the test during the first cycle (170.6% more than the element filled with polyurethane foam), while in the second cycle the element that absorbs more energy is filled with Multitek (139.0% more than the element filled with Elastopack-201).

The results obtained in this experimental campaign will allow us to advance in the next phase of the investigation. A FEM (finite elements method) numerical model was prepared to analyze the quantitative contribution of different parameters in hybrid tubes: steel tubes’ diameters and thicknesses, yield strength of different types of steel, properties of the core materials, and adhesion behavior of the filling material to the steel tubes. The possibilities are so numerous that it is virtually impossible to experimentally explore all the combinations of these design parameters. However, this set of experimental results, presented here for a limited number of cases, are important indeed for fitting the numerical model and rendering a model connected to the actual behavior of the hybrid tubes. The results of this ongoing research will be published elsewhere. Additionally, an analytical study will be carried out using Eurocodes 3 and 4 that will complement the experimental results and the simulations.

## 6. Conclusions

This article shows the results of an experimental investigation carried out on the compression behavior of hybrid steel tubes formed by two concentric steel tubes and four different fillers of nonmetallic material interposed between the two tubes: low-density polyurethane foam, two-component polyurethane grout (Elastopack^®^-201), two-component thixotropic epoxy (Multitek^®^) and one-component cement-based mortar (SikaGrout^®^-295). The polyurethane foam filled tube, which behaves most similarly to the unfilled double steel tube, reached a maximum load in the first cycle of 263.59 kN and did not buckle after this maximum peak. On the other hand, the tubes with the other fillers displayed a different behavior, which was shown through two load cycles with the following characteristics:

1. In the first cycle, the maximum load was 221.02 kN for Elastopack-201, 279.49 kN for Multitek (26.5% increase), and 268.21 kN for SikaGrout-295 (21.4% increase). After this first maximum load, the load decreased to 100, 170 and 200 kN, respectively. This decrease in load was due to the crushing process at the ends of the tubes during compression and did not result in a loss of stability due to buckling.
2. In the second cycle, the maximum load was 202.18 kN in the tube with Elastopack-201, 305.97 kN in the case of Multitek (51.3% increase), and 290.18 kN with the SikaGrout-295 filler (43.5% increase). Once this maximum load was reached, the tubes filled with Elastopack-201 and Multitek failed by buckling, while the tube filled with SikaGrout-295 maintained its vertical directrix until the end of the test and did not fail by buckling, although it had significant deformations in the upper zone of load application.

As such, the incorporation of a resistant filler into the double tube improved its mechanical behavior by allowing for a second load cycle (avoiding buckling failure at the end of the first cycle). Likewise, although the initial slopes of the load-displacement curve were similar in the three hybrid tubes (as only the steel tubes operated in that first phase), the remainder of the load-displacement curve showed specific behavior in each case that responded to the intrinsic characteristics of the filler used. Thus, for example, the lowest ultimate load obtained during the first cycle with the Elastopack-201 filler was due to its elastomeric structure, which allowed it to deform significantly after a load was applied. As the compressive load on the element increased, the core material compressed and generated increasing lateral reactions of the core against the tube walls, resulting in a loss of its load capacity (even less than that of the polyurethane foam-filled tube). However, Multitek (epoxy) and SikaGrout-295 (cement-based mortar) fillers have very high elastic moduli and are therefore capable of absorbing significant loads without significant deformation. As such, these fillers did not cause significant lateral thrusts during compression and contributed to the increase in the load capacity of the hybrid tube (this effect was more pronounced in the SikaGrout-295 filler, which maintained stability throughout the test and did not fail due to buckling).

Furthermore, the evaluation of the technical efficiency of the three fillers by analyzing the load/weight ratio of the tubes has led to the conclusion that the Multitek-filled tube had the best ratios, both in the first cycle (3.24 kN/N with a 13.3% increase compared to SikaGrout-295 filler) and in the second cycle (3.54 kN/N with a 31.6% increase compared to Elastopack-201 filler). However, when evaluating economic efficiency by means of the load–cost ratio, this filler proves to be unsuitable, and the SikaGrout-295-filled tube is much more cost-effective (12.21 kN/€ and 13.21 kN/€ for the first and second cycles, respectively).

The strain energy, a consequence of the internal energy consumed during the test, is also an indicator of the degree of ductility of each hybrid tube and, therefore, of its structural integrity. As such, the experimental results obtained in the tests have led to the conclusion that the SikaGrout-295 filler absorbs more strain energy per unit of weight than the rest of the fillers during the first cycle (27.46 J/N compared to values around 15 J/N). However, if the second load cycle (until the second maximum load is reached) is also taken into account, the Multitek filler absorbs more strain energy than the other fillers (96.10 J/N compared to 66.15 J/N for SikaGrout-295 and 46.17 J/N for Elastopack-201).

Taking into account all the parameters analyzed, Multitek filling is considered a very suitable option since it presents a good technical compression behavior (maximum loads, load–weight ratios, and absorbed energy), especially in those cases where light and resistant structures are required because a reduced weight is relevant and can offset the higher cost compared to SikaGrout-295 filling.

In summary, the experimental work carried out has shown that the use of non-metallic fillers in double steel tube structures improves their compression behavior and confers the technical performance characteristic of the filler material used.

Therefore, this type of hybrid tubular structure provides a wider range of characteristics (due to the wide variety of materials and thicknesses that can be selected for tubes and fillers) that will allow the adaptation of its structural performance to the technical specifications required in each specific application.

## Figures and Tables

**Table 1:** Characteristics of the hybrid tubular elements studied.

Length L (mm)	Tubes Steel	Core Material	Core Mat. Density ρn (kg/m^3^)	Core Mat. Elastic Mod. E (GPa)	Core Mat. Strength fc (MPa)	Inner/Outer Tube Diam. D_1_/D_2_ (mm)	Inner/Outer Tube Thickness t_1_/t_2_ (mm)
900	S-275 JR	Polyurethane foam ^1^	---	---	---	80/60	2/2
900	S-275 JR	Elastopack	1060	0.01	9.00	80/60	2/2
900	S-275 JR	Multitek	1800	2.9	90.00	80/60	2/2
900	S-275 JR	SikaGrout-295	2300	37.0	84.50	80/60	2/2

^1^ The mechanical properties of this core material are negligible with respect to the global behavior of the tubes.

**Table 2:** Results obtained during the tests in each cycle.

	Axial Load (kN)		Axial Displacement (mm)		Time (s)	
Material	N_1,max_	N_2,max_	δ_1,max_	δ_2,max_	t_1,max_	t_2,max_
Polyurethane foam	263.59	---	6.39	---	39.20	---
Elastopack-201	221.02	202.18	7.64	26.06	47.20	157.00
Multitek	279.49	305.97	8.04	38.42	49.10	231.40
SikaGrout-295	268.21	290.18	13.06	28.42	79.20	171.30

**Table 3:** Results obtained during the tests in each of the cycles (maximum and minimum) and the load efficiency coefficients η11, η22 and η12.

	Cycle 1		Cycle 2		Force Efficiency		
	N_1,max_ (kN)	N_1,min_ (kN)	N_2,max_ (kN)	N_2,min_ (kN)	η11 (N_1,min_/N_1,max_)	η22 (N_2,min_/N_2,max_)	η12 (N_1,max_/N_2,max_)
Polyurethane foam	263.59	140.48	--	--	0.53	--	--
Elastopack-201	221.02	102.61	202.18	41.36	0.46	0.20	1.09
Multitek	279.49	163.24	305.97	100.85	0.58	0.33	0.91
SikaGrout-295	268.21	196.3	290.18	156.96	0.73	0.54	0.92

**Table 4:** The weight and cost values of the hybrid elements and the ratio of the values obtained in the tests in each of the cycles (N_1,max_ y N_2,max_) with respect to these.

	Weight (N)	Cost (Eur)	N_1,max_ (kN)	N_2,max_ (kN)	N_1,max_/ Weight (kN/N)	N_2,max_/ Weight (kN/N)	N_1,max_/ Cost (kN/Eur)	N_2,max_/ Cost (kN/Eur)
Polyurethane foam	59.22	18.11	263.59	--	4.45	--	14.55	--
Elastopack-201	75.22	38.49	221.02	202.18	2.94	2.69	5.74	5.25
Multitek	86.38	140.93	279.49	305.97	3.24	3.54	1.98	2.17
SikaGrout-295	93.93	21.97	268.21	290.18	2.86	3.09	12.21	13.21

**Table 5:** Values obtained for the absolute strain energy (E_a_) and the strain energy relative to weight (E_ar_) in cycles 1 and 2 with the values of displacement (δ) and time (t) at each of the maximum loads of each cycle.

	δ_1,max_ (mm)	t_1,max_ (s)	E_a1_ (J)	E_ar1_ (J/N)	δ_2,max_ (mm)	t_2,max_ (s)	E_a2_ (J)	E_ar2_ (J/N)
Polyurethane foam	6.39	39.00	953.17	16.09	--	--	--	--
Elastopack-201	7.04	47.20	1072.09	14.25	26.06	157.00	3472.59	46.17
Multitek	8.04	49.10	1298.41	15.03	38.42	231.40	8301.76	96.10
SikaGrout-295	13.06	79.20	2579.56	27.46	28.42	171.30	6213.68	66.15

**Table 6:** Percentage difference in maximum load, the load–weight ratio of the tubes and the strain energy during the test.

	Maximum Load				Load–Weight Ratio				Strain Energy			
	Cycle 1		Cycle 2		Cycle 1		Cycle 2		Cycle 1		Cycle 2	
	N_1,max_ (kN)	∆%	N_2,max_ (kN)	∆%	N_1,max_/Weight (kN/N)	∆%	N_2,max_/Weight (kN/N)	∆%	E_a1_ (J)	∆%	E_a2_ (J)	∆%
Polyurethane f.	263.59	19.2	--	-	4.45	55.6	--	-	953.17	-	--	-
Elastopack-201	221.02	-	202.18	-	2.94	2.8	2.69	-	1072.09	12.5	3472.59	-
Multitek	279.49	26.5	305.97	51.3	3.24	13.3	3.54	31.6	1298.41	36.2	8301.76	139.0
SikaGrout-295	268.21	21.4	290.18	43.5	2.86	-	3.09	14.9	2579.56	170.6	6213.68	78.9
